# IL‐17A Promotes NETs Formation via the PKCζ–ERK–ROS–PAD4 Pathway in a Mouse Model of Ischemic Stroke

**DOI:** 10.1002/cns.70825

**Published:** 2026-03-10

**Authors:** Chang Liu, Qi Chen, Weijia Chen, Xinyue Han, Xiaowen Qiao, Jun Guo, Wei Su, Qingqing Dai

**Affiliations:** ^1^ Department of Geriatrics, Beijing Tsinghua Changgung Hospital, School of Clinical Medicine Tsinghua University Beijing China

**Keywords:** IL‐17A, ischemic stroke, NETs, PAD4

## Abstract

**Aims:**

Interleukin‐17A (IL‐17A) aggravates poststroke neurological damage and enhances neutrophil extracellular traps (NETs) formation, yet the underlying mechanism remains unclear. This study aimed to elucidate how IL‐17A regulates NETs generation after ischemic stroke.

**Methods:**

Using a mouse middle cerebral artery occlusion (MCAO) model, we administered the Peptidylarginine deiminase 4 (PAD4) inhibitor GSK484 or an IL‐17A‐neutralizing antibody (IL‐17mAb). Infarct volume and neurological function were assessed, and protein expression of PAD4, myeloperoxidase (MPO), citrullinated histones H3 (CitH3), protein kinase Cζ (PKCζ), and phosphorylated extracellular signal‐regulated kinase (p‐ERK) was evaluated. IL‐17A^−^/^−^ mice and primary neutrophils were used to further validate the signaling pathway.

**Results:**

Inhibiting PAD4 with GSK484 can significantly reduce infarct size, improve neurological outcomes, and decrease PAD4, MPO, and CitH3 protein levels. Subsequently, we found that IL‐17mAb treatment can reduce the expression of PAD4, MPO, and CitH3 in the peri‐infarct area, as well as the expression of PKC‐ζ and p‐ERK. Similarly, the expression of PAD4 and CitH3 decreased in the peri‐infarct area of IL‐17A^−^/^−^ MCAO mice. Finally, we verified IL‐17A induced PAD4 upregulation via the PKCζ–ERK–ROS axis in vitro.

**Conclusion:**

IL‐17A promotes NETs formation by upregulating PAD4 through the PKCζ–ERK–ROS pathway, exacerbating ischemic brain injury. Targeting this axis may offer a novel therapeutic strategy for stroke.

## Graphical abstract

Our study demonstrates that IL‐17A promotes NET formation by upregulating PAD4 through the PKCζ–ERK–ROS pathway, thereby exacerbating ischemic brain injury. IL‐17A regulates NET formation through this newly identified molecular pathway, and targeting this pathway may offer a novel therapeutic strategy for stroke.

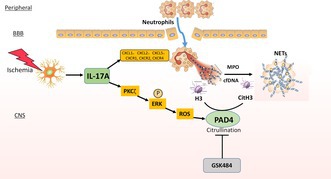

## 1 Introduction

Stroke is a leading cause of morbidity, mortality, and disability worldwide [1]. Currently, the only approved treatments for acute ischemic stroke involve recanalization therapies, including mechanical thrombectomy and intravenous thrombolysis with recombinant human plasminogen activator (rt‐PA). Although the time window for recanalization therapy has been extended from 3 h to 4.5 h, the recanalization rate was only 30% [2, 3]. Therefore, it is important to explore the new neuroprotection target.

Neutrophil extracellular traps (NETs) are web‐like structures composed of DNA scaffolds embedded with more than 30 proteins and enzymes, including histones, myeloperoxidase (MPO), and neutrophil elastase (NE) [4]. Recently, the NETs were discovered in the thrombus of patients with ischemic stroke [5] and are closely related to the neurological prognosis of patients with ischemic stroke [6, 7]. Further studies indicate that the release of NETs is not only related to the formation of thrombus, but also participates in the inflammatory response of brain tissue after ischemic stroke [8, 9, 10].

Peptidylarginine deiminase 4 (PAD4) is mainly expressed in granulocytes and monocytes and is the only enzyme in the family that has nuclear localization properties and acts on nuclear histones. It can mediate the deimination of histones H3 into citrullinated histones H3 (CitH3), release MPO and NE from azurophilic bodies in the cytoplasm, and then translocate into the nucleus, causing chromatin decondensation, nuclear membrane rupture, and ultimately the formation of NETs [11, 12]. CitH3 as a biomarker for NETs formation was reported to be associated with venous thromboembolism in cancer patients [13] and impaired wound healing in diabetes [14]. Besides, a positive correlation existed between the 24‐h improvement in NIHSS scores and the difference in CitH3 levels in between the cerebral infarct site and femoral artery. Treatment with the PAD4 inhibitor reduced NET formation and improved neurological outcomes in stroke‐affected mice [15]. Despite growing evidence implicating NETs in ischemic stroke pathology, the upstream regulatory mechanisms controlling PAD4 activation in the ischemic brain remain poorly understood.

Interleukin‐17A (IL‐17A) is a key cytokine of the IL‐17 family that plays a pivotal role in host defense and tissue inflammation [16]. Studies have shown that IL‐17A can recruit neutrophils to the central nervous system through chemokine receptors and aggravate brain ischemia in mice [17, 18]. Recent studies have found IL‐17A could regulate NETs formation [19, 20, 21], while the precise molecular mechanisms remain unclear.

In this study, we demonstrate that IL‐17A promotes NETs formation by regulating PAD4 activation via the protein kinase Cζ (PKCζ)—phosphorylated extracellular signal‐regulated kinase (p‐ERK)—reactive oxygen species (ROS) signaling pathway in ischemic stroke. Understanding this pathway may provide novel insights into therapeutic strategies targeting NETs for stroke treatment.

## 2 Methods

### 2.1 Preparation of the Mouse Middle Cerebral Artery Occlusion (MCAO) Model

Adult male C57BL/6J mice aged 6–8 weeks and weighing 18–22 g were used for animal experiments. 1% sodium pentobarbital (0.07 g/kg) was intraperitoneally injected for anesthesia. A longitudinal incision of about 1 cm was made along the midline of the neck from the mandible. The common carotid artery was then carefully freed, and the external carotid artery and the branches of the internal carotid artery were exposed to avoid damaging the blood vessels and vagus nerve. Use 5/0 black suture silk to tie a slipknot near the heart end, tie a surgical knot at the distal end of the separated external carotid artery, and place a thread at the proximal end of the external carotid artery for use. Use a micro‐artery clamp to temporarily clamp the separated internal carotid artery. Under the knot of the external carotid artery, use ophthalmic scissors to make a small incision at the bifurcation of the internal and external carotid arteries. Gently cut the external carotid artery, insert the thread plug with a head diameter of about 0.23 mm and a trunk diameter of about 0.18 mm, with the head end facing down, into the small incision, insert it in the direction of the common carotid artery, and then continue to push it proximally to the intersection of the common carotid artery, remove the artery clamp that clamps the internal carotid artery, and continue to send the thread plug along the internal carotid artery into the middle cerebral artery. After 1 h of embolization, the suture was slowly pulled out, and a double ligature was made between the small opening of the external carotid artery and the bifurcation of the internal carotid artery with suture silk, and the slipknot of the common carotid artery was untied to restore blood flow from the common carotid artery along the internal neck to the skull, and the time was recorded. The ligatures were arranged, the ends of each ligature were shortened, and then the submandibular gland was returned to its position, the incisions were aligned, the skin was sutured, and disinfected again. Note that the sham operation group only underwent surgical operations without inserting sutures. The mice were killed or perfused according to the reperfusion time required by the experiment.

### 2.2 Intraperitoneal Injection of GSK484

WT mice were divided into three groups: the Sham group, the 1 h MCAO/R 24 h group, and the 1 h MCAO/R 24 h + GSK484 group. GSK484 (#HY‐100514, Stemcell, Vancouver, CA) was dissolved in physiological saline solution and administered intraperitoneally within 3 h post‐MCAO surgery at a dose of 4 mg/kg. GSK484 dosage and administration method were chosen based on previous studies [15, 22, 23].

### 2.3 Intracerebroventricular Injections of IL‐17A‐Neutralizing mAb

Mice were randomly divided into four groups: sham, MCAO, MCAO + IgG isotype, and MCAO + IL‐17A mAb groups. The IL‐17A‐neutralizing mAb (2.0 μg, #BE0173, BioXcell, Lebanon, USA) was dissolved in InVivoPure pH 7.0 Dilution Buffer (#IP0070, BioXcell, Lebanon, USA) and injected within 3 h after MCAO, with the mouse IgG isotype injection as control (#BE0083, BioXcell, Lebanon, USA). IL‐17A mAb dosage and administration method were chosen based on previous studies [24, 25]. The intracerebroventricular injection procedure was performed as follows. Mice were anesthetized with sodium pentobarbital (70 mg/kg, i.p.) and placed in a stereotaxic frame. The cannula (28‐G, inner diameter, 0.18 mm; outer diameter, 0.36 mm) was lowered into the right cerebral ventricle using the following coordinates: 0.5 mm posterior to bregma, 1.0 mm lateral to bregma, and 3.2 mm below the skull surface.

### 2.4 Isolation and Identification of Neutrophils From Peripheral Blood of Mice

C57BL/6 mice were anesthetized and fixed on the operating table. The common carotid artery was dissected to collect 1–2 mL of peripheral blood using a heparin‐anticoagulant tube, followed by mouse euthanasia via cervical dislocation. Then red blood cells were lysed by red blood cell lysis buffer. For neutrophil sorting via BD FACSAria III flow cytometer, CD11b PE antibody (1:200, #101208, BioLegend, California, USA) and Ly6G FITC antibody (1:200, #127606, BioLegend, California, USA) were used. After incubation, the cell pellet was resuspended and filtered through a 200‐mesh strainer to remove clumps. The flow cytometer was calibrated and FITC/PE two‐parameter dot plots were employed to gate the Ly6G^+^CD11b^+^ neutrophil population. The stained cell suspension was loaded onto the cytometer for sorting. After sorting, cell purity was identified with flow cytometry and immunofluorescence assay. Ly6G FITC antibody (1:200, #127606, BioLegend, California, USA) and CD11b PE antibody (1:200, #101208, BioLegend, California, USA) were used for flow cytometry assay. Anti‐LY6G (#sc‐53,515, Santa, Santa Cruz, USA) antibody and subsequent fluorochrome‐conjugated secondary antibody (Alexa Fluor 594‐red, #ab150083, Abcam, Cambridge, UK) and DAPI were used for immunofluorescence assay.

### 2.5 Neutrophil Stimulation and Inhibitor Treatment

After isolation, neutrophils were cultured in RPMI‐1640 medium supplemented with 10% FBS and stimulated with recombinant murine IL‐17A (rmIL‐17A, 250 ng/mL, #CX14, Novoprotein, Suzhou, CN) for 1 h, 2 h, 4 h, and 6 h, respectively. The 4‐h time point was selected based on experiments indicating peak PAD4 expression at this duration. For mechanistic studies, neutrophils were pretreated for 1 h with specific inhibitors targeting PKCζ (ζ‐Stat, 10 μM) [26], ERK (Ravoxertinib, 25 nM) [27], ROS (Apocynin, 100 μM) [28], or PAD4 (GSK484, 10 μM) [29] prior to rmIL‐17A stimulation. All drugs were dissolved in DMSO (final concentration < 0.1%) and diluted in PBS. Cells were subsequently harvested for Western blot analyses.

### 2.6 Detection of ROS in Neutrophils

Dichloro‐dihydro‐fluorescein diacetate (DCFH‐DA) probe method was used according to the instructions of the Biyuntian ROS Assay Kit (S0033S). Briefly, dilute DCFH‐DA to 10 μM with serum‐free RPMI‐1640. After 24 h culture in a 96‐well plate, wash cells twice with pre‐cooled PBS, add 100 μL diluted DCFH‐DA, incubate for 20 min (shake gently every 5 min). Aspirate the probe, wash three times with pre‐cooled PBS, digest cells with 0.25% trypsin–EDTA for 2 min, and terminate with equal complete RPMI‐1640. Collect the suspension, centrifuge, and resuspend the pellet in PBS. Detect DCF fluorescence via BD FACSAria III (FSC‐A/SSC‐A gating, FITC channel: Ex 488 nm, Em 525 nm; 1 × 10^4^cells/sample, record MFI). Analyze data with FlowJo.

### 2.7 Immunofluorescence Staining

After the mice were treated according to the above grouping, the perfusion‐fixed mouse brain was frozen. According to the stereotaxic atlas of the mouse brain, frozen coronal sections were made from the rostral to the caudal side. The thickness of the brain slices was 20 μm. The cut brain slices were soaked in a 6‐well plate containing PBS and washed 3 times on a shaker, each time for 10 min. The brain slices were picked out and placed on a glass slide. The tissue to be stained was circled with a histochemical pen, soaked in PBS for 10 min, and the membrane was broken with 0.3% PBST for 30 min. The PBS was washed 3 times, each time for 5 min. Goat serum blocking solution was added, and the cells were blocked at room temperature for 1 h. The cells were washed 3 times with PBS, each time for 5 min. The diluted mixture of primary antibodies (MPO (#ab208670, Abcam, Cambridge, UK), CitH3 (#ab5103, Abcam, Cambridge, UK), and PAD4 (#ab96758, Abcam, Cambridge, UK)) was added and placed in a humidified box in a refrigerator at 4°C overnight. The next day, the slides were balanced at room temperature, washed with PBS three times for 5 min each time, incubated with fluorescent secondary antibodies (Alexa Fluor 594‐red and Alexa Fluor‐488 green) in a dark room for 30 min to 1 h at room temperature, washed with PBS three times for 5 min each time, dried, and sealed with a sealing agent containing DAPI, and photographed the next day. The expression of MPO, citH3, and PAD4 in the periphery of cerebral infarction lesions was detected.

### 2.8 Protein Immunoblotting Detection

After cells and animals are treated according to the above grouping, cell or tissue proteins are collected, quantified, and denatured and then loaded into the prepared colloid. Equal amounts of protein were loaded on SDS‐polyacrylamide gel electrophoresis gels and transferred onto polyvinylidene difluoride membranes. The primary antibodies of the target protein were used. The membranes were incubated overnight with primary antibodies. PAD4 (#ab96758, Abcam, Cambridge, UK), MPO (#ab208670, Abcam, Cambridge, UK), CitH3 (#ab5103, Abcam, Cambridge, UK), PKC‐ζ (#9368S, CST, Danvers, USA), P‐ERK (#4370S, CST, Danvers, USA), ERK (#4695S, CST, Danvers, USA), and GAPDH (#ab9485, Abcam, Cambridge, UK), followed by incubation with horseradish peroxidase‐conjugated anti‐rabbit, anti‐mouse, and anti‐rat secondary antibodies. Blots were processed using ImageJ (Java, National Institutes of Health, Baltimore, MD, USA).

### 2.9 TTC Staining to Calculate Infarct Volume

After the mice were modeled and grouped, the brains were removed by decapitation, and the tops of the brains were neatly placed in the brain mold with the top facing down. At the midpoint of the line connecting the anterior pole of the brain and the optic chiasm, the mouse brain was sliced coronally with a sharp blade; each slice was 1 mm thick, and 7 slices were cut in sequence. The sliced brain slices were placed on the blade and carefully placed in the prepared 2% 2,3,5‐Triphenyltetrazolium chloride staining (TTC) solution immediately. The staining time was about 20 min in the dark. After staining, the brain slices were placed on the glass plate in order and scanned with a scanner. The scanned images were optimized, and the infarct volume was calculated using ImageJ software. The calculation formula of brain edema rate (E) is as follows: E = (ΣVR−ΣVL)/(ΣVL + ΣVR) × 100%; ΣVR and ΣVL represent the total volume of the right cerebral hemisphere and the total volume of the left cerebral hemisphere, respectively. The background rate (B) was calculated as follows: B = TTC unstained area volume of the sham group/total volume. Percentage of cerebral infarction volume (A) = ΣSIN (1−S)/(ΣLT + ΣRT) (1−B) × 100%. ΣSIN (1−S) represents the total infarction volume after deducting the water rate.

### 2.10 Rodriguez's Neurological Function Score

From 0 to 10 points, the specific scoring principle is as follows: 0 points: no neurological function loss; 2 points: hyporesponsiveness, mild hypokinesia; 4 points: severe hypokinesia, dragging gait, lateral tilt of posture, ataxic gait, kyphosis, reduced body tone, piloerection, mild ataxia and decreased muscle strength; 6 points: body rotation, tremor/twitch/convulsion, moderate ataxia, forelimb flexion; 8 points: severe hypokinesia, respiratory distress, severe ataxia; 10 points: death occurred 72 h after reperfusion. In the above scoring criteria, neurobehavioral deficits close to the scoring criteria can be defined as 1, 3, 5, 7, and 9 points, respectively.

### 2.11 Modified Longa Score

The scoring principle of defects is as follows: 0 points: no defect; 1 point: the contralateral forelimb cannot be fully extended; 2 points: the contralateral forelimb cannot be extended; 3 points: slowly rotate to the contralateral side; 4 points: severe rotation; 5 points: fall to the contralateral side; the higher the score, the more severe the neurological defect.

### 2.12 Beam Balance Test

Before scoring, make sure the room is dark to ensure that there is no interference from external factors during the scoring process. The mouse balance beam is 120 cm long and 0.7 cm wide. The height of the crossbar from the experimental table is 40 cm. A soft foam board is laid under the crossbar to prevent the animal from falling. Mice were trained to pass the balance beam 3 days before MCAO surgery to ensure that the experimental animals could pass the balance beam within 15 s. After 3 days of training, mice that still could not pass the balance beam within 15 s were eliminated from the experiment. Neurological evaluation was according to the Beam balance test scoring improved by Feeney et al. in 1982 [30]. 6 points: The mouse can pass the balance beam without slipping. 5 points: The mouse can pass the balance beam, and the forelimb on the opposite side of the lesion slides down the beam once. 4 points: The mouse can pass the balance beam, and the forelimb on the opposite side of the lesion slides down the beam more than once, fewer than 50% of the number of steps. 3 points: The mouse can pass the balance beam, and the forelimb on the opposite side of the lesion slides down the beam more than 50% of the number of steps. 2 points: The mouse tried to pass the balance beam but failed. 1 point: The mouse remained motionless on the balance beam but was able to maintain balance. 0 points: The mouse cannot stay on the balance beam.

### 2.13 Rotating Rod Experiment

Day 1: In Acceleration (ACC)/Run mode, set ACC Time to 2 min. When the speed reaches 10 rpm, press Run to switch to constant speed mode until the total time reaches 5 min. Press stop, record time (the time the mouse is on the rod) and speed (the speed when falling), repeat three times, and the interval between each time is not less than 15 min. Day 2: A total of three trainings, the first two methods are the same as the first day, and the third one is changed to ACC mode, ACC Time is set to 300 s, the total time is 5 min, and the experiment is stopped by pressing stop. Day 3: Set the speed to 40 rpm, ACC Time to 300 s, total duration to 5 min, repeat three times. The average time of the three tests on the third day was used as the preoperative baseline level of the test mice. Behavioral test: Set the speed to 40 rpm, ACC Time to 300 s, total duration to 5 min, test each animal three times a day, record the persistence time of each animal, and the average of the three times was the score for the day.

### 2.14 Statistical Analysis of Experimental Data

Categorical variables were expressed as numbers and proportions, and continuous variables were expressed as mean ± SEM. The statistical results of basic research experimental data were analyzed using One‐way ANOVA. The followed multiple comparisons were conducted by using Bonferroni with the software GraphPad Prism version 10.5.0 for Mac (GraphPadSoftware, La Jolla, CA, USA). *p* < 0.05 was considered statistically significant.

## 3 Results

### 3.1 GSK484 Reduces Cerebral Infarction and Improves Neurological Function in MCAO Mice

**FIGURE 1:**
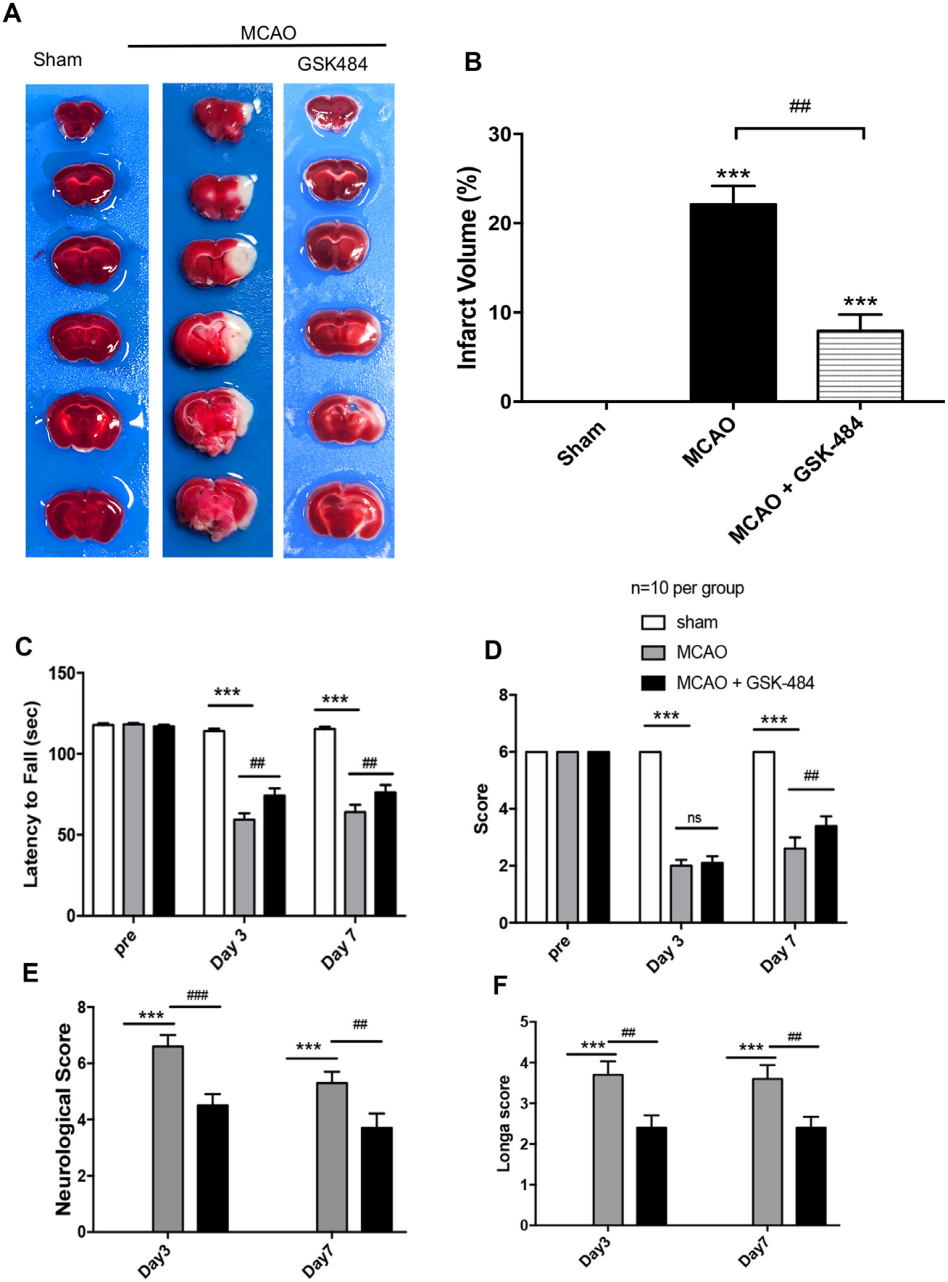
GSK484 can reduce the volume of middle cerebral artery infarction lesions in MCAO mice and alleviate neurological damage in MCAO mice. (A) A mouse model of 1 h MCAO/R for 24 h was established, and GSK484 was injected intraperitoneally at a dose of 4 mg/kg. TTC staining was used to analyze the volume of cerebral infarction (white is the infarct area, brain slice thickness is 1 mm). (B) TTC staining statistical graph, data are expressed as mean ± standard error, and statistical results were analyzed by Two‐way ANOVA, ***p < 0.001 versus sham group; ##*p* < 0.001, *n* = 6 per group. (C–F) A 1 h MCAO/R mouse model was established, and GSK484 was injected intraperitoneally. The neurological function of the mice was detected before surgery, on the 3rd day after surgery, and on the 7th day after surgery, including (C) Rota‐rod score, (D) Balance beam score, (E) neurological function score, and (F) Longa score. The data were expressed in quartiles, and the statistical results were analyzed by One‐way ANOVA. The intergroup comparison was performed using the Bonfferoni test, ***p < 0.001, ##*p* < 0.01, ###*p* < 0.001, *n* = 10 per group.

We firstly established a mouse MCAO model and administered GSK484, a PAD4 inhibitor. TTC staining revealed that GSK484 significantly reduced the cerebral infarction volume of the mice at 24 h after 1 h MCAO (Figure 1A,B). Additionally, behavioral and neurological assessments demonstrated that GSK484 could improve the neurological functional outcomes in MCAO mice, as evidenced by enhanced performance in rota‐rod, balance beam, neurological function score, and Longa score (Figure 1C–F).

### 3.2 GSK484 Suppresses PAD4, MPO, and CitH3 Expression in MCAO Mice

**FIGURE 2:**
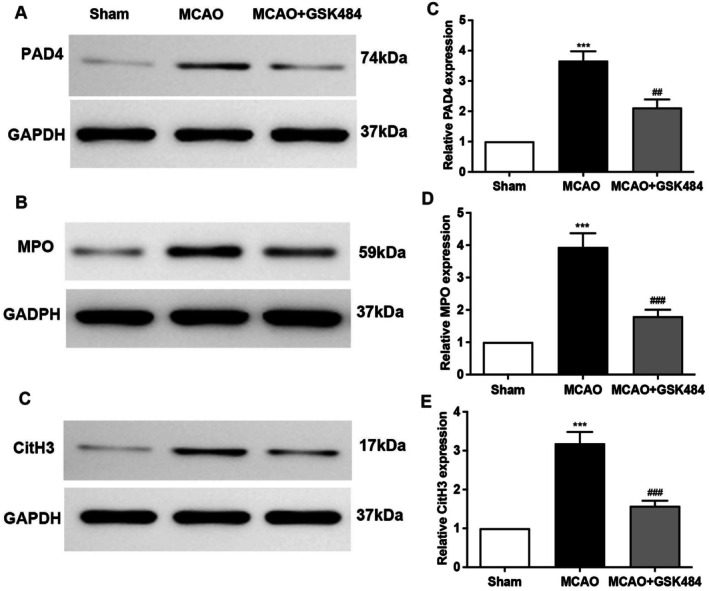
GSK484 treatment can reduce the expression of PAD4, MPO, and CitH3 proteins. The figure shows the expression of PAD4, MPO, and CitH3 proteins in the tissues of mice in the 1 h MCAO/R 24 h model, after intraperitoneal injection of GSK484. Sham is the sham operation group, control is the operation group, and GSK 484 is the group that received intraperitoneal injection of GSK484 after surgery. Figure (A–C) is a representative picture of Western blot, and Figure (C–E) is a statistical chart. Statistical results were analyzed by One‐way ANOVA. Bonfferoni test was used for comparison between groups. ***p < 0.001 compared with the Sham group, ##*p* < 0.01, ###*p* < 0.001 compared with the Control group, *n* = 6 in each group.

**FIGURE 3:**
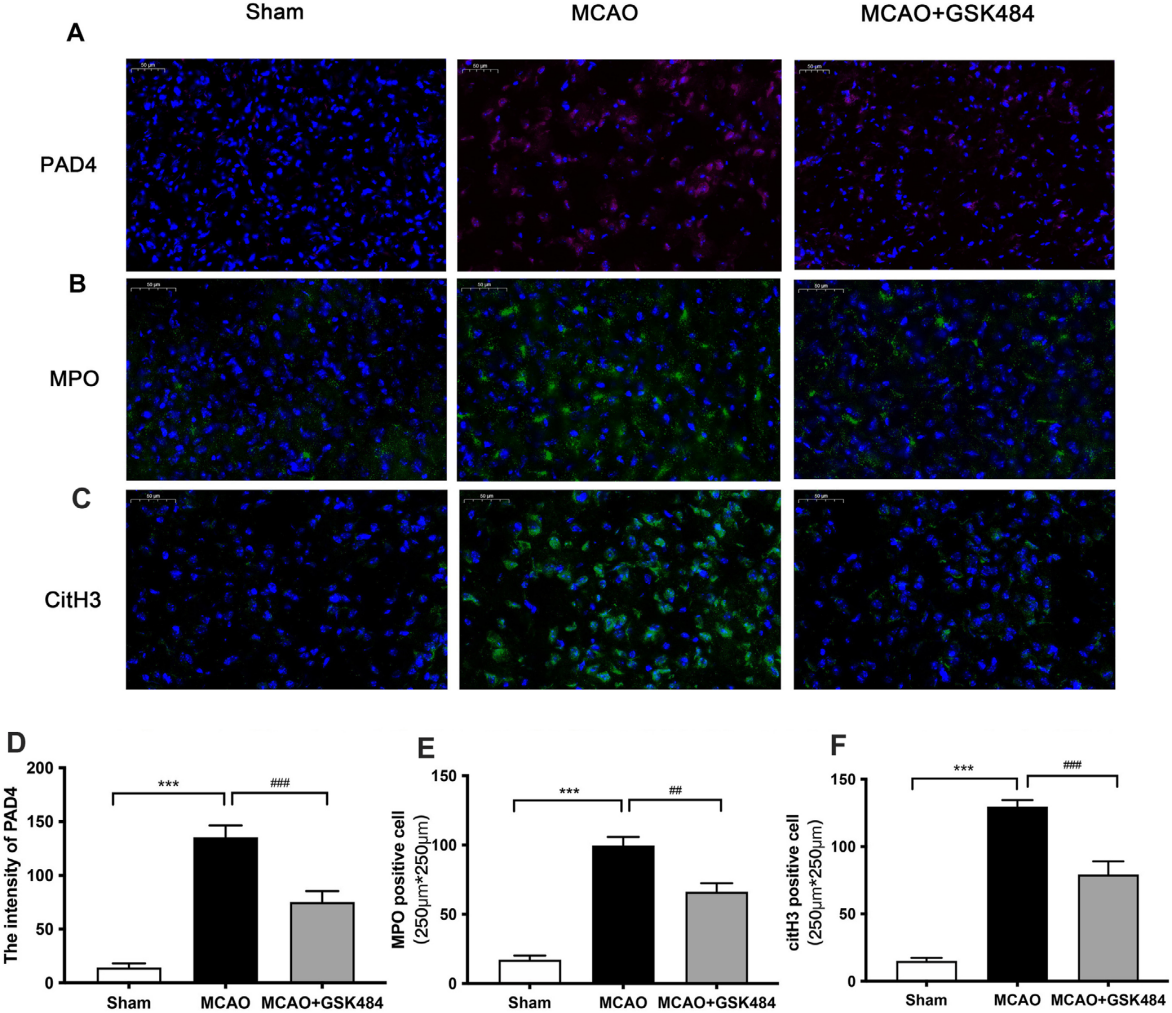
GSK484 treatment can reduce the expression of PAD4, MPO, and Cith3 proteins in the peri‐infarct area of mice after 1 h MCAO/R24h modeling. (A–C) Representative immunohistofluorescence staining images showing anti‐PAD4 (red), anti‐MPO (green), anti‐CitH3 (green), and DAPI (blue) (scale bar = 50 μm). (D–F) Statistical graph of immunohistofluorescence images. Data are expressed as mean ± standard error. Statistical results were analyzed by One‐way ANOVA. Inter‐group comparisons were performed using the Bonfferoni test. Images are representative of independent experimental batches. ***p < 0.001, ###*p* < 0.001, ##*p* < 0.01, *n* = 6 per group.

Western blot analysis of brain tissues showed that PAD4, MPO, and CitH3 protein levels were significantly elevated after MCAO but decreased following GSK484 treatment (Figure 2). Consistent with the Western blot data, Immunofluorescence staining detected elevated PAD4, MPO, and CitH3 in the peri‐infarct region of MCAO mice. While the expression of PAD4, MPO, and CitH3 was significantly reduced after treatment with GSK484 (Figure 3). These results indicate that GSK484 could effectively suppress PAD4 expression and suppress NETs formation post‐MCAO.

### 3.3 IL‐17A Neutralization Attenuates PAD4, MPO, CitH3 Expression Possibly via the PKCζ/ERK Pathway

**FIGURE 4:**
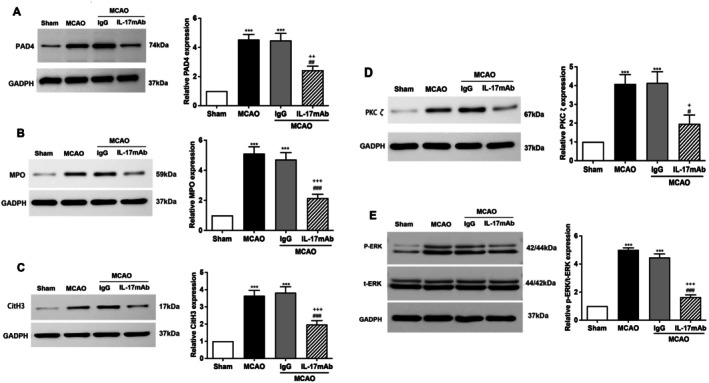
IL‐17mAb treatment can reduce the expression of PAD4, MPO, CitH3, PKC‐ζ, and P‐ERK proteins in mouse brain tissue. The figure shows the expression of PAD4 (A), MPO (B), CitH3 (C), PKC‐ζ (D), and P‐ERK (E) proteins in the brain tissues of mice treated with IL‐17mAb in the 1 h MCAO/R 24 h model. Sham is the sham operation group, control is the operation group, IgG is the isotype control antibody, and the IL‐17mAb group is IL‐17mAb treatment. The left picture is a representative picture of Western blot, and the right picture is a statistical chart. Statistical results were analyzed by One‐way ANOVA. Bonfferoni test was used for comparison between groups. ***p < 0.001 compared with the Sham group, # *p* < 0.05, ##*p* < 0.01, ###*p* < 0.001 compared with MCAO group, + *p* < 0.05, ++ *p* < 0.01, +++*p* < 0.001compared with the IgG group, *n* = 6 in each group.

To investigate whether IL‐17A regulates PAD4 expression in the MCAO model, we administered an IL‐17A‐neutralizing antibody (IL‐17AmAb). Western blot analysis showed that PAD4 protein was significantly up‐regulated after MCAO and down‐regulated after treatment with IL‐17A mAb (Figure 4A). Similarly, IL‐17A mAb inhibited the expression of MPO and CitH3 (Figure 4B,C). The results indicated that IL‐17A could promote the expression of PAD4 and the formation of NETs post‐MCAO. In 2010, Hakkim et al. used more than 1200 compounds to clarify the role of the Raf–MEK–ERK pathway in the formation of NETs, in which PAD4 can be activated by the protein kinase PKC, which is a kinase involved in ROS burst and can affect the formation of NETs [31]. We hypothesized that IL‐17A may regulate PAD4 expression through the PKCζ/ERK pathway. Indeed, the expression level of PKC‐ζ and p‐ERK was elevated post‐MCAO but decreased after IL‐17A mAb treatment (Figure 4D,E), indicating that IL‐17A may regulate PAD4 expression via the PKCζ/ERK pathway.

### 3.4 IL‐17A Deficiency Reduces PAD4 and CitH3 Expression Post‐MCAO

**FIGURE 5:**
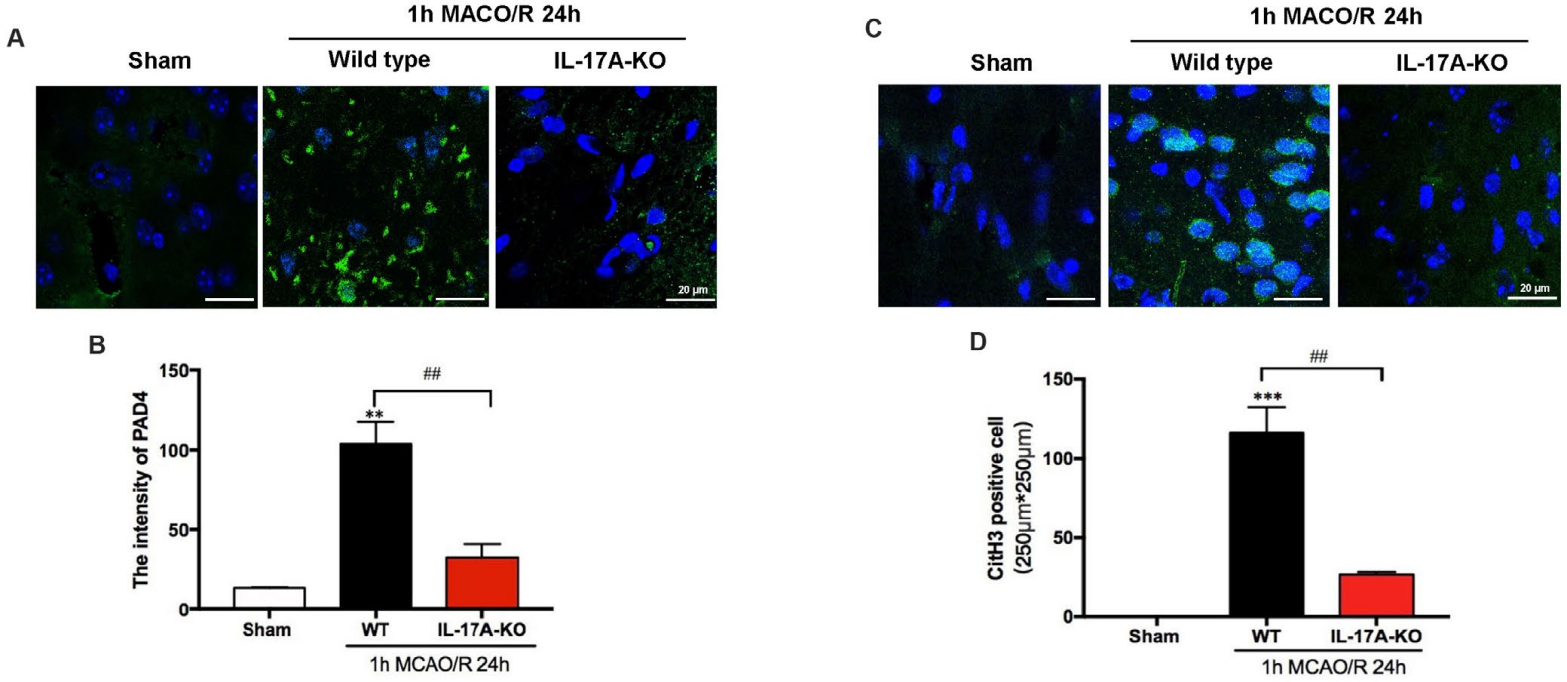
PAD4 expression and CitH3‐positive cell ratio decreased in the peri‐infarction area of IL‐17A^−^/^−^ mice. (A) Immunofluorescence staining images of the peri‐infarction area of mice. Anti‐PAD4 (green) and DAPI (blue) were used for immunofluorescence staining (scale bar = 100 μm). Green fluorescent secondary antibody was used to stain PAD4. (B) Statistical graph of PAD4 fluorescence intensity. **p < 0.01 versus sham group, ##*p* < 0.01, *n* = 6 per group. (C) Immunofluorescence staining images of the peri‐infarction area of mice. Anti‐citH3 (green) and DAPI (blue) were used for immunofluorescence staining (scale bar = 20 μm). (D) Statistical graph of CitH3‐positive cell number. Data are expressed as mean ± standard error. Statistical results were analyzed by One‐way ANOVA. Bonfferoni test was used for comparison between groups. Images are representative of independent experimental batches. ***p < 0.001 versus sham group, ## *p* < 0.01, *n* = 6 per group.

To further validate the role of IL‐17A in PAD4 activation, we analyzed IL‐17A knockout (IL‐17A^−^/^−^) mice after MCAO. Immunofluorescence staining demonstrated that the expression of PAD4 and CitH3 was significantly reduced in IL‐17A^−^/^−^ mice compared with wild‐type controls (Figure 5). This is consistent with the effects of IL‐17A mAb in Figure 4. These data collectively indicate that IL‐17A is a critical upstream regulator of PAD4‐mediated NETs formation during cerebral ischemia–reperfusion injury.

### 3.5 Pharmacological Inhibition Confirms IL‐17A‐PAD4 Signaling in Neutrophils

**FIGURE 6:**
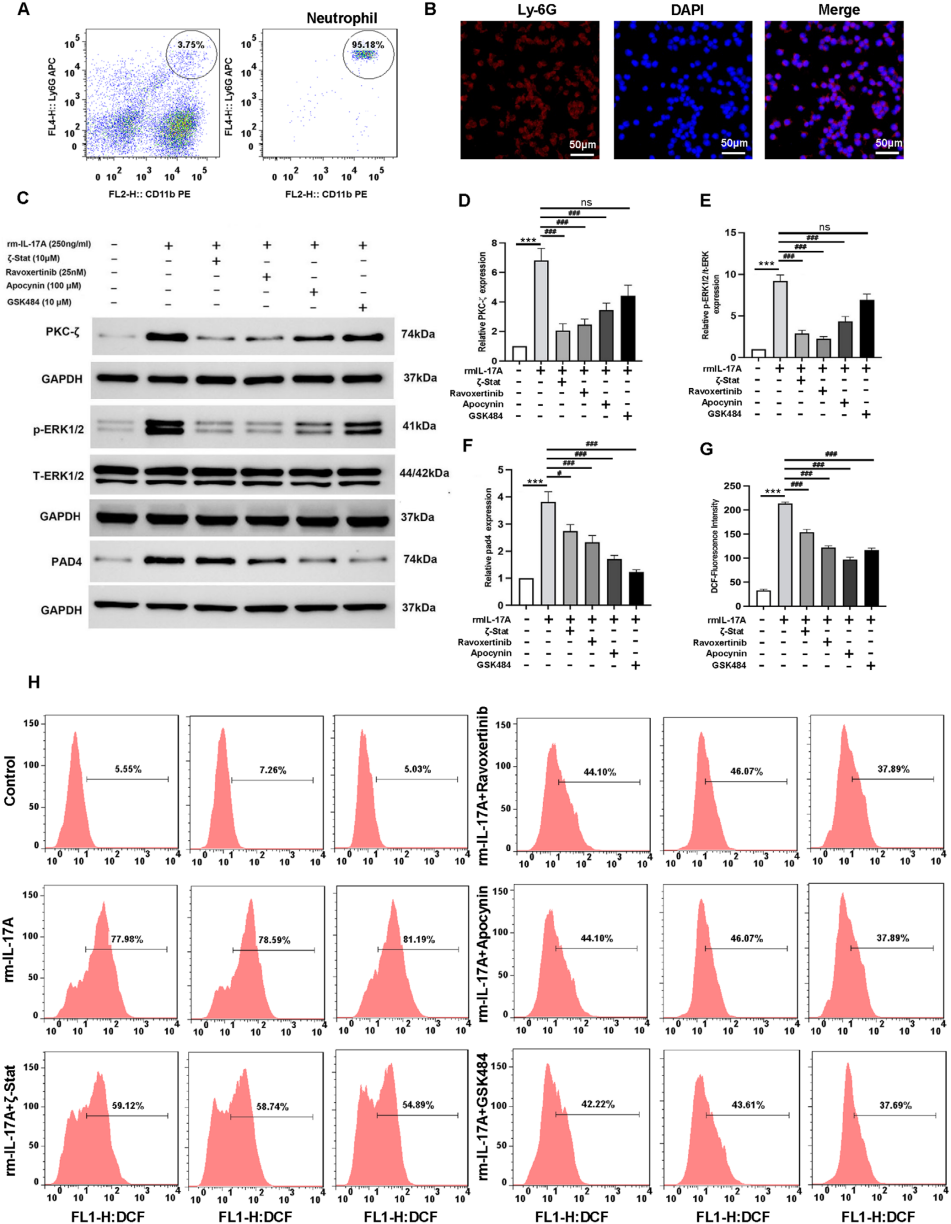
IL‐17A regulates the expression of PAD4 through PKCζ‐ERK1/2‐ROS. (A) Flow cytometry images for the sorting and identification of neutrophils using CD11b and Ly‐6G antibodies. (B) Cellular immunofluorescence of Ly‐6G showed the purity of neutrophils. (C) Primary neutrophils were taken and Western blot was used to detect the expression of target proteins under different group stimulation conditions. rm‐IL‐17A, recombinant mouse IL‐17A; ζ‐Stat, inhibitor of PKC‐ζ; Ravoxertinib, ERK kinase inhibitor; Apocynin, NADPH oxidase inhibitor; GSK484, PAD4 inhibitor. (D) Statistical graph of PCK‐ζ. (E) Statistical graph of ERK phosphorylation level. (F) Statistical graph of PAD4 expression. (G and H) The levels of ROS were detected by DCFH‐DA probe in primary neutrophils cells under different group stimulation. The statistical graph (G) and the representative flow cytometry graph (H) were presented. Statistical results were analyzed by One‐way ANOVA. Bonferroni test was used for comparison between groups, ***p < 0.001, ###*p* < 0.001, ##*p* < 0.01, #*p* < 0.05, *n* = 6 per group.

To validate the IL‐17A‐PKCζ‐ERK‐ROS‐PAD4 signaling cascade, we employed pharmacological inhibitors in primary neutrophils: ζ‐Stat (PKCζ inhibitor), ravoxertinib (p‐ERK inhibitor), apocynin (ROS inhibitor), and GSK484 (PAD4 inhibitor). Neutrophil was sorted by flow cytometry (Figure 6A) and cell purity was determinated by immunofluorescence (Figure 6B). IL‐17A stimulation consistently upregulated all pathway components (PKCζ, p‐ERK, ROS, and PAD4) (Figure 6C–H), which suggested that IL‐17A may activate this pathway. Mechanistically, we observed a stepwise signaling hierarchy: (1) PKCζ inhibition attenuated p‐ERK, ROS, and PAD4. (2) p‐ERK blockade attenuated ROS and PAD4, accompanied by a partial reduction in PKC expression. (3) ROS suppression selectively decreased expression of PAD4, accompanied by a partial reduction in PKC and ERK expression. (4) PAD4 inhibition showed no feedback effect on upstream signaling molecules PKC and ERK (Figure 6C–H). These findings demonstrate that IL‐17A may promote NETs formation by upregulating PAD4 through the PKCζ‐ERK‐ROS signaling cascade. It should be noted that this pathway is not a linear cascade but a network with multi‐node positive feedback crosstalk, which is discussed below.

## 4 Discussion

Our study identifies a previously unknown IL‐17A‐PKCζ‐ERK‐ROS‐PAD4 signaling pathway that promotes NETs formation and worsens stroke outcomes. This discovery has important clinical implications, as we demonstrate: (1) Blocking PAD4 with GSK484 significantly reduces brain damage and improves recovery; (2) IL‐17A affects NETs formation through the newly identified molecular pathway; and (3) Targeting IL‐17A (either genetically or pharmacologically) produces similar protective effects as direct PAD4 inhibition. These findings suggest new stroke treatment strategies by targeting multiple points in this pathway—IL‐17A, PKCζ, ERK, ROS, or PAD4—to block harmful NETs formation and improve recovery.

As previous studies showed that NETs play a key role in thrombosis and inflammatory responses in ischemic stroke, not only impeding recanalization but also aggravating inflammatory injury in brain tissue [8]. Elevated plasma levels of NETs have been seen in patients with ischemic stroke and are associated with poor prognosis [32]. Mice treated with NETs‐inhibitory factor had smaller brain infarcts, improved long‐term neurological and motor function, and enhanced survival after stroke [8]. Building on these clinical and preclinical observations, it established the detrimental role of NETs in stroke pathology. Our study provides direct evidence that pharmacological PAD4 inhibition with GSK484 can effectively suppress NETs formation, reduce ischemic‐infarct volume, and improve neurological function in the MCAO mice. The previous research has also shown that overexpression of PAD4 induces an increase in NETs formation that is accompanied by reduced neovascularization and increased BBB damage after stroke [10]. Genetic ablation or pharmacologic inhibition of PAD increases neovascularization and vascular repair and improves functional recovery [10]. Inhibition of NETs production by PAD4 deficiency could also restore tPA‐induced loss of blood–brain barrier integrity and consequently decrease tPA‐associated brain hemorrhage after ischemic stroke [9]. Collectively, these findings strongly support therapeutic targeting of the PAD4‐NETs axis as a promising strategy to improve outcomes in ischemic stroke by simultaneously addressing multiple pathological mechanisms. As our research centers on neutrophil extracellular trap (NETs) formation, pathological processes more directly linked to acute motor impairment than cognitive decline, we employed the rotarod test, beam balance test, modified Longa score, and Rodriguez's neurological function score to assess motor coordination, balance, limb symmetry, and overall neurological deficits respectively. Cognitive evaluations were not included.

Based on our previous findings demonstrating that intracerebroventricular injection of IL‐17A monoclonal neutralizing antibody or IL‐17A knockout mice reduced infarct volume and improved neurological prognosis in MCAO mice [25]. In the current study, we further established IL‐17A as a key regulator of PAD4 expression and NETs formation in ischemic stroke. The pathogenic role of IL‐17A extends beyond stroke pathophysiology. In the acute pneumonia mice, IL‐17A was reported to activate phosphorylated STAT3, leading to increased ROS release and promoting the formation of NETs [21], while in cancer it facilitates neutrophil recruitment and NETs generation to exclude cytotoxic T cells [19]. Notably, the study has found that IL‐17 was unable to induce pancreatitis in PAD4^−^/^−^ mice [20]. These findings indicate that IL‐17A may be an important extracellular signaling molecule that regulates PAD4 and induces the formation of NETs. Besides, studies also found that extracellular histones, a major component of NETs, trigger upregulation of IL‐17/Th17 responses and bone destruction [33], suggesting a feed‐forward loop in inflammatory diseases.

Moreover, our mechanistic investigations revealed that the PKCζ and ERK signaling pathways were activated in MCAO mice, while IL‐17A monoclonal neutralizing antibody could inhibit these signaling pathways. In vitro, we used pathway‐specific inhibitors to further reveal that IL‐17A regulates PAD4 expression and activity through the PKCζ‐ERK‐ROS signaling axis. IL‐17A signals were reported to activate the downstream transcription factor C/EBPβ through the transcription factor NF‐κB, transcription activator AP‐1, and ERK [34]. The formation of NETs depends on the generation of ROS [35, 36]. PKCζ is an activator of PAD4 with the possible mechanism that PKCζ assists in activating the NADPH oxidase complex [37, 38]. PLC/PKC/ERK signaling pathway was also reported to be involved in NETs formation [39]. Previous research has shown that IL‐17A promotes ROS release and NETs formation via STAT3 activation [21]. We characterize a previously unrecognized PKCζ‐ERK‐ROS‐PAD4 signaling cascade through which IL‐17A promotes NETs formation, revealing an additional layer of regulation in neutrophil‐driven ischemic brain injury.

Notably, our mechanistic studies revealed that the IL‐17A‐PKCζ‐ERK‐ROS‐PAD4 pathway is not a linear cascade but a network with multi‐node positive feedback crosstalk, which explains the observed changes in upstream proteins following downstream inhibitor treatment. First, ERK and ROS function as feedback amplifiers: Activated ERK stabilizes PKCζ via phosphorylation and transcriptional regulation [39], while ROS enhances PKCζ activation through oxidative modification of cysteine residues and inhibits its dephosphorylation [37]; ROS also stabilizes ERK phosphorylation by suppressing negative regulators such as MKP‐1 [36]. Thus, inhibition of ERK or ROS blocks their positive feedback on upstream molecules, leading to reduced PKCζ or ERK expression. Secondly, a reciprocal feedback loop exists between PAD4 and ROS: As demonstrated by DCF fluorescence assays (Figure 6G,H), PAD4 inhibition with GSK484 significantly reduced ROS levels. This is because PAD4‐mediated NETs formation releases CitH3 and MPO, which activate the NADPH oxidase complex to promote ROS production [29]; additionally, PAD4 inhibits ROS‐scavenging enzymes to maintain ROS stability [36]. Collectively, these feedback loops are physiologically meaningful for amplifying inflammatory signals during ischemic stroke, ensuring efficient NETs formation to participate in pathological processes. Importantly, PAD4 inhibition did not affect the core expression of PKCζ or ERK (Figure 6D,E), indicating that feedback regulation is localized to specific nodes rather than disrupting the entire cascade. These observations are consistent with previous reports on kinase signaling crosstalk in NETs formation and ischemic injury [37, 39], further validating the authenticity and complexity of the pathway identified in our study. This multilayered regulation also suggests that targeting feedback nodes (e.g., ROS or ERK) may synergistically inhibit NETs formation by blocking both forward signaling and amplifying loops, providing a potential strategy for optimizing stroke therapy.

Although rt‐PA and mechanical thrombectomy remain the primary recanalization treatments for acute ischemic stroke, their efficacy is limited, partly due to NETs‐mediated thrombus resistance. Our results indicate that inhibiting NETs formation by targeting IL‐17A or PAD4, as well as its signaling pathways, may effectively reduce NETs formation, thereby enhancing thrombolysis efficacy and reducing inflammatory injury in brain tissue. Future research should further investigate the clinical potential and safety of these interventions.

This study has some limitations. First, we primarily used the MCAO/R mice and in vitro experiments, without further validation in other stroke models. NETs formation is a complex process, and other molecular mechanisms involved in NETs formation beyond PAD4 warrant further exploration. Additionally, the exact role of IL‐17A in ischemic stroke remains to be clarified to develop more targeted therapeutic strategies. Future studies could expand to other stroke models, validate the role of PAD4 and IL‐17A under various conditions, and optimize drug dosages and timing to better guide clinical applications.

In conclusion, this study demonstrates that IL‐17A regulates PAD4 and NETs formation via the PKC‐ζ‐ERK‐ROS signaling axis, offering new therapeutic targets for ischemic stroke. This discovery provides a promising foundation for the development of combination therapeutic strategies targeting IL‐17A and PAD4, potentially offering an effective approach for the treatment of ischemic stroke.

## Funding

This work was supported by the National Natural Science Foundation of China, 82101535; Beijing Tsinghua Changgung Hospital Fund, 12025C01005; The Beijing High‐Level Innovation and Entrepreneurship Talent Support Program young backbone talent projects, G202531272.

## Ethics Statement

All procedures were performed in accordance with the recommendations in the Guide for the Care and Use of Laboratory Animals of the National Institutes of Health and approved by the experimental animal ethics committee of the Capital Medical University (SCXK2021‐0006).

## Conflicts of Interest

The authors declare no conflicts of interest.

## Data Availability

The data that support the findings of this study are available on request from the corresponding authors.
